# A Note on Radiative Heat Transfer to Peristaltic Flow of Sisko Fluid

**DOI:** 10.1155/2015/283892

**Published:** 2015-02-10

**Authors:** Obaid Ullah Mehmood, Constantin Fetecau

**Affiliations:** ^1^Department of Mathematics, COMSATS Institute of Information Technology, Wah Cantt. 47040, Pakistan; ^2^Department of Mathematics, Technical University of Iasi, 70050 Iasi, Romania; ^3^Academy of Romanian Scientists, 050094 Bucuresti, Romania

## Abstract

This paper looks at the effects of radiative heat transfer on the peristaltic transport of a Sisko fluid in an asymmetric channel with nonuniform wall temperatures. Adopting the lubrication theory, highly nonlinear coupled governing equations involving power law index as an exponent have been linearized and perturbation solutions are obtained about the Sisko fluid parameter. Analytical solutions for the stream function, axial pressure gradient, axial velocity, skin friction, and Nusselt number are derived for three different cases (i.e., shear thinning fluid, viscous fluid, and shear thickening fluid). The effects of Grashof number, radiation parameter, and other configuration parameters on pumping, trapping, temperature, Nusselt number, and skin friction have been examined in detail. A good agreement has been found for the case of viscous fluid with existing results.

## 1. Introduction

Naturally inherited mechanism of peristalsis has been accorded to attention due to its enormous implications in physiological and industrial applications. Peristalsis is a natural phenomenon responsible for the transportation and digestion of food in the living bodies [1]. It is controlled by the involuntary sinusoidal displacements running along the tract boundaries and pumps the contents from the area of lower pressure to area of higher pressure [2]. This mechanism has marvelously revolutionized the industrial applications as well. Most of the realistic physiological and industrial fluids are non-Newtonian in nature as mentioned by the experimental study of Joseph [3], for instance, food bolus in oesophagus, chyme in stomach and intestines, reproductive and glandular secretions, and flow of metal alloys in automobiles and machines and many others. Peristalsis in presence of heat transfer is imperative in many realistic cases like oxygenation, hemodialysis, perfusion of arterial-venous blood, metabolic processes, external heat sources, and others. Radiative heat transfer is very important in the treatment of diseased tissues in cancer. Few recent investigations on peristaltic flow with heat transfer include the work through the studies [4–11].

A numerical investigation on the peristaltic motion of Sisko fluid in an asymmetric channel is done by Wang et al. [12]. Further, Nadeem and Akbar [13] considered the problem of peristaltic flow of Sisko fluid in an inclined uniform tube. Again, Nadeem et al. [14] made an analytical and numerical study of peristaltic flow of Sisko fluid in an endoscope. Recently, Shafie et al. [15] studied thermal diffusion and diffusion thermoeffects on the flow of Sisko fluid in nonuniform channel. More recently, Mehmood et al. [16] investigated the influences of dissipative heating and partial slip on the peristaltic flow of Sisko fluid. To the best of our knowledge, no study is yet reported on the radiative heat transfer analysis of peristaltic flow of Sisko fluid. In vivo rheology reveals that during various radioactive treatments, peristaltic flows of biofluids undergo through a variety of thermal radiations. The investigation of these issues will provide a profound understanding of peristaltic rheology in more realistic situations. The main purpose of this paper is to study the effects of radiative heat transfer on the peristaltic transport of a Sisko fluid. The flow is considered in an asymmetric channel having nonuniform wall temperatures. Present problem results into a system of highly nonlinear coupled partial differential equations involving power law index as an exponent. The derivation of solutions for these complex equations strengthens the novel contribution of the present work. Sisko fluid model is considered for present analysis because, for different values of material parameters, it represents different models which are Newtonian, shear thinning, or shear thickening fluid models. Governing equations are solved adopting the long wavelength and low Reynolds number approximations and using the regular perturbation method. Due to the involvement of power law index *n* in the governing equations, it is impossible to find the general solutions which are valid for all values of *n*. Therefore, the analytical solutions are obtained for three particular cases which correspond to shear thinning fluid for *n* = 0, viscous fluid for *n* = 1, and shear thickening fluid for *n* = 2. Adopting a similar approach the solutions for other values of *n* can also be obtained. It is also important to mention that our limiting solutions for Newtonian fluids are identical to those obtained by Mishra and Ramachandra Rao [17]. Finally, the results are graphically presented and discussed for various pertinent parameters.

## 2. Problem Development

**Figure 1:**
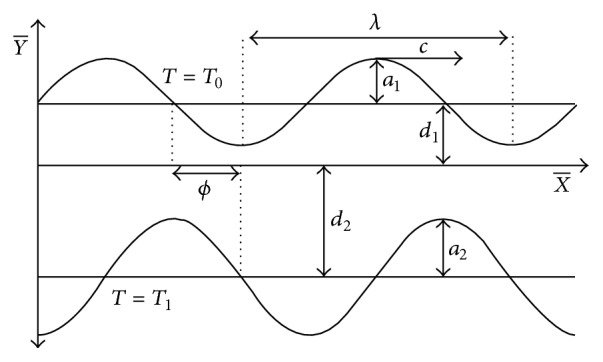
Sketch of the physical model.

Two-dimensional flow of an incompressible Sisko fluid in an asymmetric channel is considered. The flow is engendered by sinusoidal peristaltic waves travelling with constant speed *c* along the walls of the channel. The Cartesian coordinate system $\left(\bar{X},\bar{Y}\right)$ is chosen in such a way that $\bar{X}$-axis lies along the center of the channel while $\bar{Y}$-axis is transverse to it. The sketch of the physical model is given in Figure 1. The sinusoidal shapes of the upper and lower walls of the channel are modeled by [17]

(1) $$\begin{array}{c} {\bar{H}}_{1}\left(\bar{X},\bar{t}\right)=d_{1}+a_{1}\cos\left\{\frac{2\pi }{\lambda }\left(\bar{X}-c\bar{t}\right)\right\},\\ {\bar{H}}_{2}\left(\bar{X},\bar{t}\right)=-d_{2}-a_{2}\cos\left\{\frac{2\pi }{\lambda }\left(\bar{X}-c\bar{t}\right)+\phi \right\}, \end{array}$$

where *a*_*i*_ (*i* = 1,2) are the upper and lower wave amplitudes, *d*_*i*_ (*i* = 1,2) are the upper and lower channel widths, *λ* is the wavelength, $\bar{t}$ is the time and *ϕ* is the phase difference with 0 ≤ *ϕ* ≤ *π*, and  *ϕ* = 0 gives the waves out of phase while for *ϕ* = *π* the waves are in phase. Further, *a*_*i*_, *d*_*i*_, and *ϕ* are related by *a*_1_^2^ + *a*_2_^2^ + 2*a*_1_ *a*_2_cos⁡*ϕ* ≤ (*d*_1_ + *d*_2_)^2^ [17]. The radiative heat is induced to the flow by maintaining the upper and lower walls of the asymmetric channel at nonuniform temperatures *T*_0_ and *T*_1_, respectively.

The continuity, momentum, and energy equations modeling the two-dimensional flow of a Sisko fluid are

(2) ∂U−∂X−+∂V−∂Y−=0,ρ∂∂t−+U−∂∂X−+V−∂∂Y−U−  =−∂P−∂X−+∂S−X− X−∂X−+∂S−X− Y−∂Y−+ρgβT−T0,ρ∂∂t−+U−∂∂X−+V−∂∂Y−V−=−∂P−∂Y−+∂S−X− Y−∂X−+∂S−Y− Y−∂Y−,ρξ∂∂t−+U−∂∂X−+V−∂∂Y−T=k∂2T∂X−2+∂2T∂Y−2+∂Q¯∂Y¯.

Here ${\overset{-}{S}}_{\bar{X}\;\bar{X}}$, ${\overset{-}{S}}_{\bar{X}\;\bar{Y}}$, and ${\overset{-}{S}}_{\bar{Y}\;\bar{Y}}$ are the nontrivial components of the extra stress tensor $\bar{S}$ corresponding to Sisko fluid defined by [18]

(3) $$\begin{array}{c} \bar{S}=\left[{\bar{a}}_{s}+{\bar{b}}_{s}{\left|\sqrt{\frac{1}{2}\mathrm{tr}({\bar{A}}^{2})}\right|}^{n-1}\right]\bar{A},\\ \bar{A}=\text{grad}\;V+{\left(\text{grad}\;V\right)}^{T}, \end{array}$$

**V** is the velocity vector, and the radiative flux $\partial \bar{Q}/\partial \bar{Y}$ has the form [19]

(4) $$\begin{array}{c} \frac{\partial \bar{Q}}{\partial \bar{Y}}=4\alpha^{2}\left(T-T_{0}\right). \end{array}$$

In the above expressions *ρ* is the fluid density, $\bar{P}$ is the pressure, *g* is the gravitational acceleration, *β* is the coefficient of thermal expansion, *k* is the thermal conductivity, *ξ* is the specific heat at constant volume, *α* is the mean radiation absorption coefficient, *T* is the temperature of fluid, $\bar{U}$ and $\bar{V}$ are the velocity components in axial and transverse directions, respectively, and ${\bar{a}}_{s}$, ${\bar{b}}_{s}$ and *n* are material parameters. For ${\bar{a}}_{s}=0$ the generalized power law model is obtained and for ${\bar{a}}_{s}=\mu$ (*μ* is the dynamic viscosity) and ${\bar{b}}_{s}=0$ the Newtonian fluid model is recovered. Further, at the channel walls the no-slip conditions are taken into account.

The flow is unsteady in the laboratory frame $\left(\bar{X},\bar{Y}\right)$. However, we consider the flow in the wave frame $\left(\bar{x},\bar{y}\right)$ moving with constant wave speed *c* where the flow becomes steady. The flow quantities in the two frames are related by the following transformations:

(5) $$\begin{array}{c} \bar{x}=\bar{X}-c\bar{t},\;\;\bar{y}=\bar{Y},\;\;\bar{u}=\bar{U}-c,\\ \bar{v}=\bar{V},\;\;\bar{p}=\bar{P}, \end{array}$$

where $\left(\bar{u},\bar{v}\right)$ are the axial and transverse velocity components and $\bar{p}$ is the pressure in the wave frame. Using the transformations (5) along with the nondimensional quantities

(6) x=2πλx¯,  y=y¯d1,  u=u¯c,  v=v¯c,h1=H¯1d1,  h2=H¯2d1,  p=2πd12a¯sλcp¯,S=d1a¯scS¯,  δ=2πd1λ,Re=ρcd1a¯s,  bs=b¯sa¯sd1/cn−1,  η=T−T0T1−T0,Gr=ρgβd12T1−T0a¯sc,  Pe=ρcd1ξk,  N2=4α2d12k,

letting the stream function *ψ* related to the velocity components *u* and *v* by

(7) $$\begin{array}{c} u=\frac{\partial \psi }{\partial y},\;\;v=-\delta \frac{\partial \psi }{\partial x}, \end{array}$$

and utilizing the long wavelength approximation, (2) are reduced to

(8) $$\begin{array}{c} \frac{\partial^{4}\psi }{\partial y^{4}}+b_{s}\left\{\frac{\partial^{2}}{\partial y^{2}}\left({\left|\frac{\partial^{2}\psi }{\partial y^{2}}\right|}^{n}\right)\right\}+\text{Gr}\frac{\partial \eta }{\partial y}=0, \end{array}$$

(9) $$\begin{array}{c} \frac{dp}{dx}=\frac{\partial^{3}\psi }{\partial y^{3}}+b_{s}\left\{\frac{\partial }{\partial y}\left({\left|\frac{\partial^{2}\psi }{\partial y^{2}}\right|}^{n}\right)\right\}+\text{Gr}\;\eta , \end{array}$$

(10) $$\begin{array}{c} \frac{\partial^{2}\eta }{\partial y^{2}}+N^{2}\eta =0, \end{array}$$

where Gr is Grashof number and *N* is the radiation parameter. The appropriate boundary conditions are

(11) $$\begin{array}{c} \psi =\frac{F}{2},\;\frac{\partial \psi }{\partial y}=-1,\;\eta =0,\;\text{at}\;\;y=h_{1}\left(x\right),\\ \psi =-\frac{F}{2},\;\frac{\partial \psi }{\partial y}=-1,\;\eta =1,\;\text{at}\;\;y=h_{2}\left(x\right), \end{array}$$

with wall shapes *h*_1_(*x*) and *h*_2_(*x*) in dimensionless form as

(12) $$\begin{array}{c} h_{1}\left(x\right)=1+a\cos\left(x\right),\;\;h_{2}\left(x\right)=-d-b\cos\left(x+\phi \right), \end{array}$$

where *a* = *a*_1_/*d*_1_, *b* = *a*_2_/*d*_1_, and *d* = *d*_2_/*d*_1_ are configuration parameters satisfying the relation *a* ^2^ + *b* ^2^ + 2*ab*cos⁡*ϕ* ≤ (1 + *d*)^2^. The dimensionless volume flow rate *F* in the wave frame is defined by

(13) $$\begin{array}{c} F=\overset{h_{1}(x)}{\underset{h_{2}(x)}{\int }}\frac{\partial \psi }{\partial y}dy=\psi \left(x,h_{1}\left(x\right)\right)-\psi \left(x,h_{2}\left(x\right)\right) \end{array}$$

and is related to the dimensionless time mean flow rate *θ* in laboratory frame by *F* = *θ* − 1 − *d*.

## 3. Perturbation Solution

Equation (10) along with the corresponding boundary conditions (11) is directly solved by integration. The solutions that have been obtained for temperature *η* and Nusselt number Nu are

(14) $$\begin{array}{c} \eta =\frac{\sin N\left(h_{1}-y\right)}{\sin N\left(h_{1}-h_{2}\right)},\\ \text{Nu}=\frac{N\cos N\left(h_{1}-y\right)}{\sin N\left(h_{1}-h_{2}\right)}. \end{array}$$

Equations (8) and (9) are highly nonlinear and coupled and cannot be directly solved. Therefore, we seek the perturbation solutions and express the flow quantities into series expansions by taking Sisko fluid parameter *b*_*s*_ as perturbation parameter. These expansions are

(15) $$\begin{array}{c} \psi =\overset{\infty }{\underset{i=0}{\sum }}b_{s}^{i}\psi_{i},\;\;p=\overset{\infty }{\underset{i=0}{\sum }}b_{s}^{i}p_{i},\\ u=\overset{\infty }{\underset{i=0}{\sum }}b_{s}^{i}u_{i},\;\;\tau =\overset{\infty }{\underset{i=0}{\sum }}b_{s}^{i}\tau_{i}. \end{array}$$

Invoking series expansions (15) into (8) and (9) with boundary conditions (11), we obtain the zeroth and first order systems. Since these systems involve the power law index *n* as an exponent, general solutions which are valid for all values of *n* cannot be calculated. Thus, by fixing the values of power law index *n* for three particular cases, namely, *n* = 0, *n* = 1, and *n* = 2, the solutions for the stream function *ψ*, axial pressure gradient *dp*/*dx*, axial velocity *u*, and skin friction *τ* are given in the next three subsections.

### 3.1. Case  I (*n* = 0 Shear Thinning Fluids)

The solutions corresponding to this case are

(16) $$\begin{array}{c} \psi =A_{1}+A_{2}y+A_{3}y^{2}+A_{4}y^{3}+\frac{\text{Gr}\;\cos\;N\left(h_{1}-y\right)}{N^{3}\sin N\left(h_{1}-h_{2}\right)},\\ \frac{dp}{dx}=6A_{4},\\ u=A_{2}+2A_{3}y+3A_{4}y^{2}+\frac{\text{Gr}\;\sin\;N\left(h_{1}-y\right)}{N^{2}\sin N\left(h_{1}-h_{2}\right)},\\ \tau =2A_{3}+6A_{4}y-\frac{\text{Gr}\;\cos\;N\left(h_{1}-y\right)}{N\sin N\left(h_{1}-h_{2}\right)}+b_{s}. \end{array}$$

### 3.2. Case  II (*n* = 1)

The solutions corresponding to this case are

(17) ψ=A1+A2y+A3y2+A4y3+Gr cos⁡ Nh1−yN3sinNh1−h2+bsGrcos⁡Nh1−yN3sinNh1−h2C1+C2y+C3y2+C4y3   −Gr cos⁡ Nh1−yN3sinNh1−h2,

(18) $$\begin{array}{c} \frac{dp}{dx}=6A_{4}+b_{s}\left\{6A_{4}+C_{4}\right\}, \end{array}$$

(19) u=A2+2A3y+3A4y2+Gr sin N(h1−y)N2sinN(h1−h2)+bsC2+2C3y+3C4y2−Gr sin N(h1−y)N2sinN(h1−h2),

(20) τ=2A3+6A4y−Gr cos⁡ Nh1−yNsinNh1−h2+bs2A3+2C3+6A4y+6C4y.

Note that for ${\bar{a}}_{s}=\mu$ and *b*_*s*_ = 0  (17)–(20) give just the solutions for Newtonian fluids. Further, for Gr = 0 and = 0 ,  (17) and (18) reduce to the forms

(21) ψ=F+h1−h2h2−h132y3−3h1+h2y2+6h1h2y−y1h2−h13F2+h1h23−3h1h22       −h2−F2h13−3h2h12,dpdx=−121h1−h22+Fh1−h23,

which are identical to those obtained by Mishra and Ramachandra Rao [17, Equations (7) and (9)].

### 3.3. Case  III (*n* = 2 Shear Thickening Fluids)

The solutions corresponding to this case are

(22) ψ=A1+A2y+A3y2+A4y3+Gr cos⁡ Nh1−yN3sinNh1−h2+bsG1+G2y+G3y2+G4y3+G5y4  +G6cos⁡Ny+G7ycos⁡Ny+G8cos⁡2Ny  +G9sinNy+G10ysinNy+G11sin2NyG4y3+G5y4,dpdx=6A4+bs64A3A4+G4,u=A2+2A3y+3A4y2+Gr sin Nh1−yN2sinNh1−h2+bsG2+2G3y+3G4y2+4G5y3  +G7+NG9cos⁡Ny+NG10ycos⁡Ny  +2NG11cos⁡2Ny+G10−NG6sinNy  −NG7ysinNy−2NG8sin2Ny3G4y2+4G5y3,τ=2A3+6A4y−Gr cos⁡ Nh1−yNsinNh1−h2+bs2N2sinNh1−h2−1Gr2+2N22A32+G3+12A3A4y+3G4y1−cos⁡2Nh1−h2Gr2+2N22A32+G3+12A3A4y+3G4y   ·1−cos⁡2Nh1−h2Gr2+2N22A32+G3+12A3A4y+3G4y1−cos⁡2Nh1−h2   ·2N2sinNh1−h2−1.

All coefficients appearing into the above expressions are calculated by usual lengthy algebra that is involved in the regular perturbation method. Here, the pressure rise Δ*P*_*λ*_, skin friction *τ*, and Nusselt number Nu are obtained by the relations [6]

(23) $$\begin{array}{c} \Delta P_{\lambda }=\overset{2\pi }{\underset{0}{\int }}\frac{dp}{dx}dx,\;\;\tau =S_{xy},\;\;\text{Nu}=-\frac{\partial \eta }{\partial y}. \end{array}$$

## 4. Results and Discussion

The purpose of this section is to describe the influence of various interesting parameters (i.e., Grashof number Gr, radiation parameter *N*, amplitude ratio *a*, channel width ratio *d*, and phase difference *ϕ*) on the pressure rise Δ*P*_*λ*_, axial pressure gradient *dp*/*dx*, axial velocity *u*, streamline patterns, temperature *η*, Nusselt number Nu, and skin friction *τ*. Figures 3–9 present the effects of these parameters on the flow.

### 4.1. Validation

**Figure 2:**
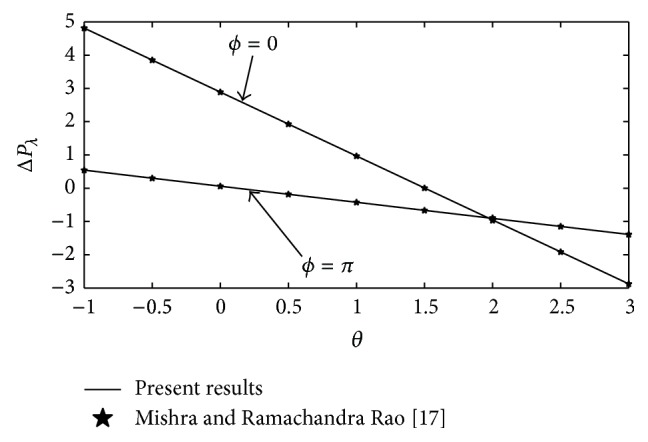
Comparison of pressure rise Δ*P*_*λ*_ against flow rate *θ* with Mishra and Ramachandra Rao's results [17] when *d* = 2, *a* = 0.7, *b* = 1.2, *b*_*s*_ = 0, *n* = 1, Gr = 0, and *N* = 0.

To assure the validity of the present study, the Sisko fluid parameter *b*_*s*_, Grashof number Gr, and radiation parameter *N* are taken zero and our solutions are reduced to the particular case of viscous fluid. The results for the pressure rise Δ*P*_*λ*_ are compared in Figure 2 with the known results of Mishra and Ramachandra Rao [17] and a very good agreement is observed.

### 4.2. Pumping

**Figure 3:**
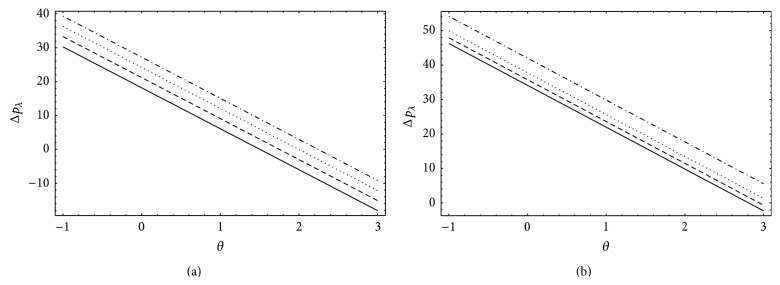
Pressure rise Δ*p*_*λ*_ for *a* = 0.7, *b* = 1.2, *d* = 2, *ϕ* = 0, *n* = 2, and *b*_*s*_ = 0.01: (a) *N* = 1, _—_Gr = 0, _– – –_Gr = 1, _…_Gr = 2, _._._._Gr = 3 and (b) Gr = 5, _—_ *N* = 0.1, _– – –_ *N* = 0.3, _…_ *N* = 0.4, and _._._._ *N* = 0.5.

**Figure 4:**
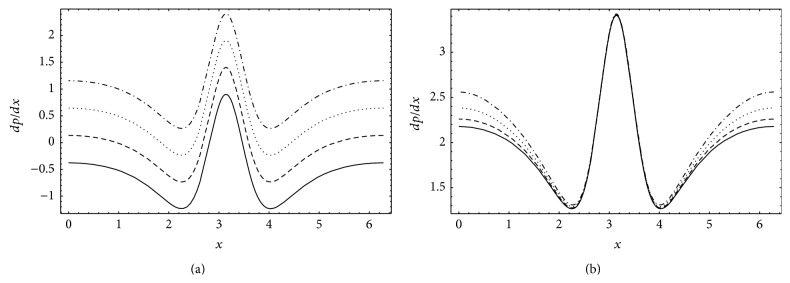
Axial pressure gradient *dp*/*dx* for *a* = 0.7, *b* = 1.2, *d* = 2, *ϕ* = 0, *b*_*s*_ = 0.01, *n* = 2, and *θ* = 1.8: (a) *N* = 0.1, _—_Gr = 0, _– – –_Gr = 1, _…_Gr = 2, and _._._._Gr = 3 (b) Gr = 5, _—_ *N* = 0.10, _– – –_ *N* = 0.15, _…_ *N* = 0.20, and _._._._ *N* = 0.25.

This subsection describes the effects of Gr and *N* on pumping phenomenon by presenting the plots in Figures 3 and 4. Figures 3(a) and 3(b) elucidate the variations in pressure rise Δ*P*_*λ*_ for different Gr and *N*, respectively. It is noticed that retrograde, peristaltic, free, and augmented pumping increases with increasing Gr and *N*. Physically, this shows that the buoyancy effects and heat radiation increase the pumping efficiency. Moreover, an inverse relationship is found between the pressure rise Δ*P*_*λ*_ and the flow rate *θ*; that is, Δ*P*_*λ*_ decreases as *θ* increases. The effects of Gr and *N* on the axial pressure gradient *dp*/*dx* are displayed in Figures 4(a) and 4(b), respectively. *dp*/*dx* increases with increasing Gr and *N* and the variations are more significant near the inlet and outlet of the wave. Further, *dp*/*dx* reaches it maximum value at the center of the wave.

### 4.3. Velocity

**Figure 5:**
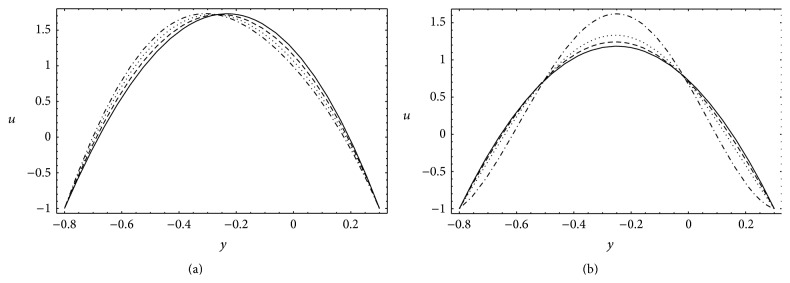
Axial velocity *u* for *a* = 0.7, *b* = 1.2, *d* = 2, *ϕ* = 0, *b*_*s*_ = 0.01, *n* = 2, *x* = *π*: (a) *N* = 5, *θ* = 2.2, _—_Gr = 0, _– – –_Gr = 2, _…_Gr = 4, _._._._Gr = 6 and (b) Gr = 5, *θ* = 1.8, _—_ *N* = 0.0, _– – –_ *N* = 2.5, _…_ *N* = 2.7, _._._._ *N* = 2.8.

Figure 5 portrays the variations of transverse distribution of the axial velocity for different values of Gr and *N*. Present analysis shows that in wave frame the axial velocity satisfies the no-slip boundary conditions (*u* = −1) at both lower (*y* = *h*_2_) and upper (*y* = *h*_1_) channel walls. The dispersion in the axial velocity is dominant near the centerline of the channel.  Figure 5(a) shows that the velocity *u* increases below the centerline and decreases above it for increasing Gr. Physically, the buoyancy effects accelerate the flow below the centerline and decelerate it above the centerline.  Figure 5(b) displays the influence of *N* on *u*. It is clearly seen that *u* decreases in the neighborhood of channel walls and increases near the centerline if *N* increases. Due to the heat radiation the fluid gains velocity in the centerline while it loses velocity near the channel walls.

### 4.4. Trapping

**Figure 6:**
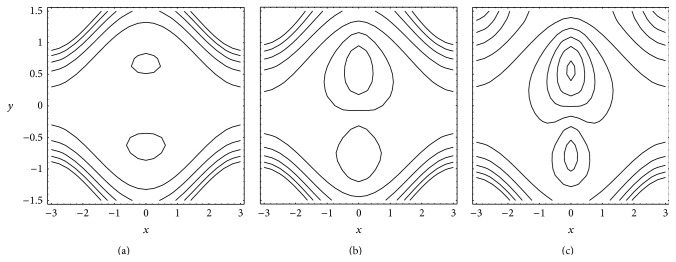
Streamlines for different Gr (a) 0, (b) 3 and (c) 9 with fixed *a* = 0.5, *b* = 0.5, *d* = 1, *ϕ* = 0, *N* = 1, *θ* = 1, *n* = 2, and *b*_*s*_ = 0.01.

**Figure 7:**
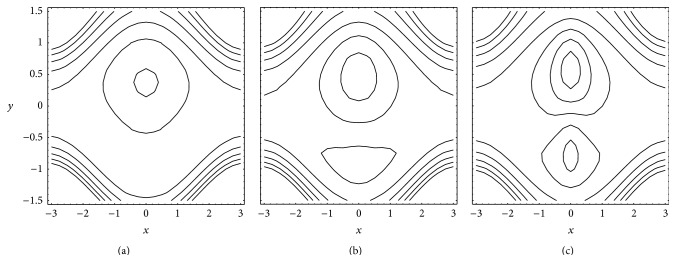
Streamlines for different *N* (a) 0.1, (b) 0.9, and (c) 1.0 with fixed *a* = 0.5, *b* = 0.5, *d* = 1, *ϕ* = 0, Gr = 5, *θ* = 1.1, *n* = 2, and *b*_*s*_ = 0.01.

Figures 6 and 7 present the streamlines for different values of Gr and *N*, respectively. From Figure 6 it results that in absence of buoyancy effects (Gr = 0) the trapping is almost symmetric about the centerline. An increase in buoyancy effects disturbs this symmetry. In the presence of buoyancy effects (Gr ≠ 0) the trapping becomes dominant near the upper wall. Further, trapping increases with an increase in Gr. It is seen from Figure 7 that for small *N* trapping exists near the upper wall only. But for large *N* trapping near the lower wall is also noticed. Further, trapping increases with an increase in *N*.

### 4.5. Temperature

**Figure 8:**
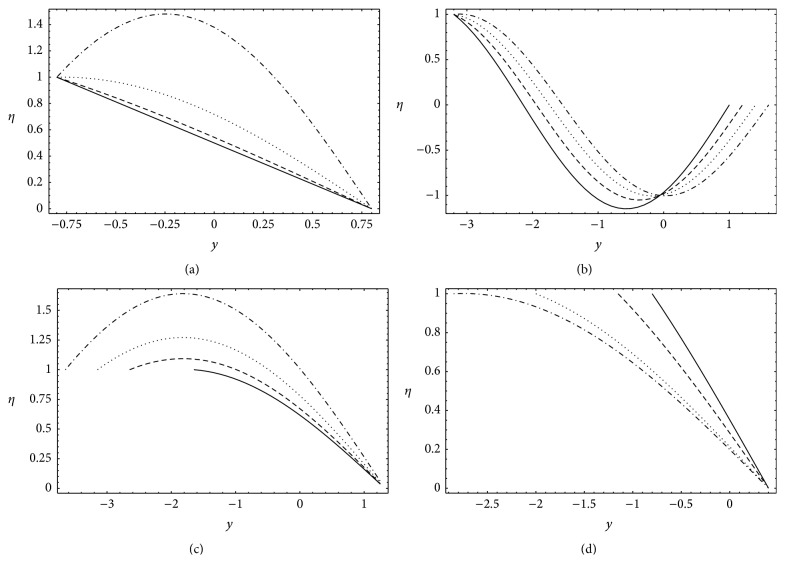
Temperature profiles *η* for *b* = 1.2, *b*_*s*_ = 0.01, and *n* = 2: (a) *a* = 0.2, *d* = 2, *x* = *π*, *ϕ* = 0, _—_ *N* = 0.0, _– – –_ *N* = 0.5, _…_ *N* = 1.0, and _._._._ *N* = 1.5, (b) *d* = 2, *x* = 0, *ϕ* = 0, *N* = 1, _—_ *a* = 0.0, _– – –_ *a* = 0.2, _…_ *a* = 0.4, and _._._._ *a* = 0.6, (c) *a* = 0.6, *x* = 1, *ϕ* = 0, *N* = 0.5, _—_ *d* = 1.0, _– – –_ *d* = 2.0, _…_ *d* = 2.5, and _._._._ *d* = 3.0, and (d) *a* = 0.6, *d* = 2, *x* = *π*, *N* = 0.5, _—_ *ϕ* = 0*π*/4, _– – –_ *ϕ* = *π*/4, _…_ *ϕ* = 2*π*/4, and _._._._ *ϕ* = 3*π*/4.

Figure 8 present the effects of the radiation parameter *N*, amplitude ratio *a*, channel width ratio *d*, and phase difference *ϕ* on the temperature profiles *η*. It is found that significant variations in the temperature profiles occur in the center of the channel and temperature profiles, as expected, are almost parabolic. Moreover, the temperature *η* clearly satisfies the boundary conditions at upper (*η* = 0 at *y* = *h*_1_) and lower (*η* = 1 at *y* = *h*_2_) walls of the channel.

In Figure 8(a) the temperature profiles of *η* for different values of *N* have been plotted. In absence of heat radiation (*N* = 0) the temperature profile is linear while for *N* ≠ 0 the temperature profile is parabolic. Further, the temperature increases with increasing *N*. This increase becomes sharp for large values of *N*. Figures 8(b) and 8(c) are plotted for different *a* and *d*. They depict that *η* increases with increasing *a* and *d*. Further, Figure 8(d) reveals the decreasing nature of *η* with respect to *ϕ*.

### 4.6. Nusselt Number and Skin Friction

**Figure 9:**
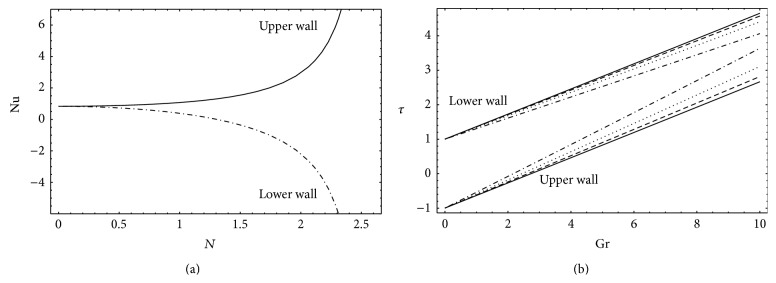
(a) Nusselt number for *a* = 0.6, *b* = 1.2, *d* = 2, *ϕ* = 0, *b*_*s*_ = 0.01, and *x* = *π*, (b) skin friction for *a* = 0.7, *b* = 1.2, *d* = 2, *ϕ* = 0, *θ* = 1.8, *b*_*s*_ = 0.01, *x* = *π*/4, *n* = 2, _—_ *N* = 0.1, _– – –_ *N* = 0.2, _…_ *N* = 0.3, and _._._._ *N* = 0.4.

In Figure 9(a) the Nusselt number Nu is plotted against radiation parameter *N* at both upper (*y* = *h*_1_) and lower (*y* = *h*_2_) walls. It is noted that the increase in *N* enhances Nu at the upper wall and decreases it at the lower wall. Skin friction *τ* is sketched versus Grashof number Gr for different *N* at both upper (*y* = *h*_1_) and lower (*y* = *h*_2_) walls in Figure 9(b). This figure shows that *τ* is an increasing function of Gr. *τ* also increases at the upper wall and decreases at the lower wall when *N* is increased.

## 5. Conclusion

The problem of radiative heat transfer with nonuniform wall temperatures on peristaltic flow of the Sisko fluid in an asymmetric channel is studied and the following interesting points have been noticed.

(i) Buoyancy and radiation effects enhance the pumping and trapping phenomena.
(ii) Buoyancy effects on velocity profiles show opposite behaviors below and above the centerline. The radiation parameter also shows opposite behavior on velocity near walls and centerline.
(iii) Temperature increases with increasing radiation parameter, amplitude ratio, and channel width ratio.
(iv) Nusselt number increases at the upper wall and decreases at the lower wall when radiation parameter increases.
(v) Skin friction increases when Grashof number increases and shows opposite behaviors at lower and upper walls with regard to the radiation parameter.
(vi) Our results, corresponding to *b*_*s*_ = Gr = *N* = 0, are in good agreement with those of Mishra and Ramachandra Rao [17].
